# Supplementary material for: Geometric transformation adaptive optics (GTAO) for volumetric deep brain imaging through gradient-index lenses
Source: Nat Commun. 2024 Feb 3;15:1031. doi: 10.1038/s41467-024-45434-5 (PMC10838304; doi:10.1038/s41467-024-45434-5)
Supplement: Supplementary file 1 — Supplementary information [file 41467_2024_45434_MOESM1_ESM.pdf]

Supplementary Material for

Geometric transformation adaptive optics (GTAO) for volumetric deep brain imaging through gradient-index lenses

Yuting Li[1, 2]†, Zongyue Cheng[1, 2]†, Chenmao Wang[1, 2], Jianian Lin[1, 2], Hehai Jiang[1, 2] and Meng Cui[1, 2, 3*]

[1]School of Electrical and Computer Engineering, Purdue University, West Lafayette, IN 47907, USA

[2]Bindley Bioscience Center, Purdue University, West Lafayette, IN 47907, USA

[3]Department of Biology, Purdue University, West Lafayette, IN 47907, USA

†These authors contributed equally to this work.

*Correspondence:

Dr. Meng Cui,

(+1) 765-496-1332

Email: mengcui@purdue.edu

This file includes Supplementary Figures 1-8.

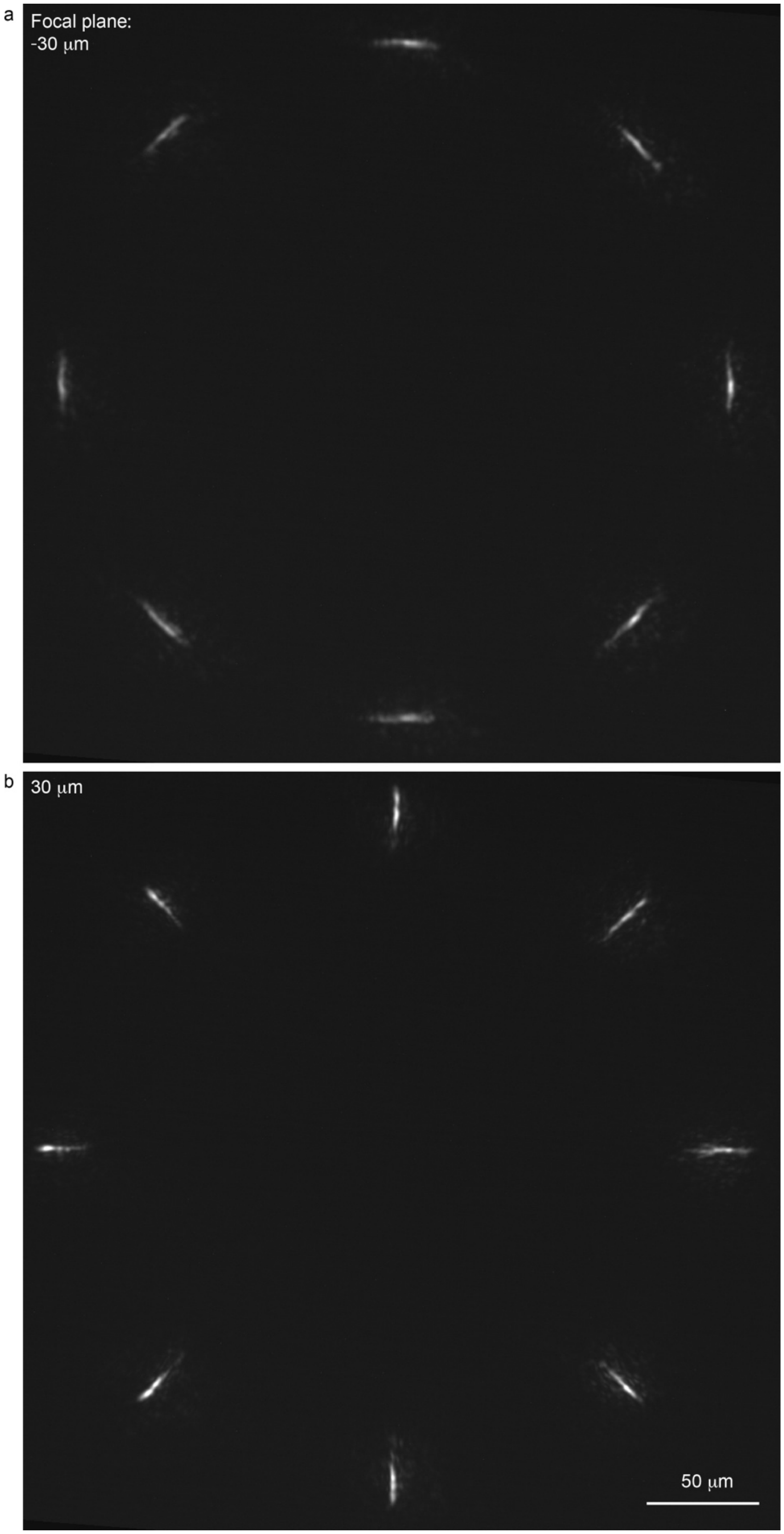

Supplementary Figure 1 | Focal profiles through 0.5 mm diameter 1.5 pitch 6.7 mm long GRIN lens at two planes spaced by 60 microns. The eight foci were 140 microns away from the optical axis with an angular spacing of 45 degrees. (a) Tangential focal plane. (b) Sagittal focal plane. The imaging experiment was repeated independently 2 times with similar results.

(a) Table of aberration coefficients.

**Zernike Coefficients Uncorrected**	**Zernike Coefficients Corrected**	**j**	**Zernike Polynomial $Z_j(\rho, \theta)$**	**Aberration**
-0.00001187	-0.01034903	5	$\sqrt{6}\rho^2 \sin(2\theta)$	Primary astigmatism at 45°
-0.95650143	0.01225403	6	$\sqrt{6}\rho^2 \cos(2\theta)$	Primary astigmatism at 0°
0.00000219	-0.02052655	7	$\sqrt{8}(3\rho^3 - 2\rho)sin\theta$	Primary y coma
0.01306444	0.00724213	8	$\sqrt{8}(3\rho^3 - 2\rho)cos\theta$	Primary x coma
-0.00000069	-0.06563088	9	$\sqrt{8}\rho^3 \sin(3\theta)$	
-0.06711070	-0.06626744	10	$\sqrt{8}\rho^3 \cos(3\theta)$	
-0.03759695	-0.04735100	11	$\sqrt{5}(6\rho^4 - 6\rho^2 + 1)$	Primary spherical
0.02225823	0.00264508	12	$\sqrt{10}(4\rho^4 - 3\rho^2)\cos(2\theta)$	Secondary astigmatism at 0°
0.00000021	-0.00232457	13	$\sqrt{10}(4\rho^4 - 3\rho^2)\sin(2\theta)$	Secondary astigmatism at 45°
-0.00172994	0.00103271	14	$\sqrt{10}\rho^4 \cos(4\theta)$	
-0.00000004	-0.01470172	15	$\sqrt{10}\rho^4 \sin(4\theta)$	
-0.00153786	-0.00197576	16	$\sqrt{12}(10\rho^5 - 12\rho^3 + 3\rho)cos\theta$	Secondary x coma
0.00000028	0.00003305	17	$\sqrt{12}(10\rho^5 - 12\rho^3 + 3\rho)sin\theta$	Secondary y coma
0.00146188	0.00186694	18	$\sqrt{12}(5\rho^5 - 4\rho^3)\cos(3\theta)$	
0.00000002	0.00153084	19	$\sqrt{12}(5\rho^5 - 4\rho^3)\sin(3\theta)$	
-0.00007174	0.00073927	20	$\sqrt{12}\rho^5 \cos(5\theta)$	
-0.00000001	-0.00157862	21	$\sqrt{12}\rho^5 \sin(5\theta)$	
-0.00043696	-0.00024351	22	$\sqrt{7}(20\rho^6 - 30\rho^4 + 12\rho^2 - 1)$	Secondary spherical

(b)

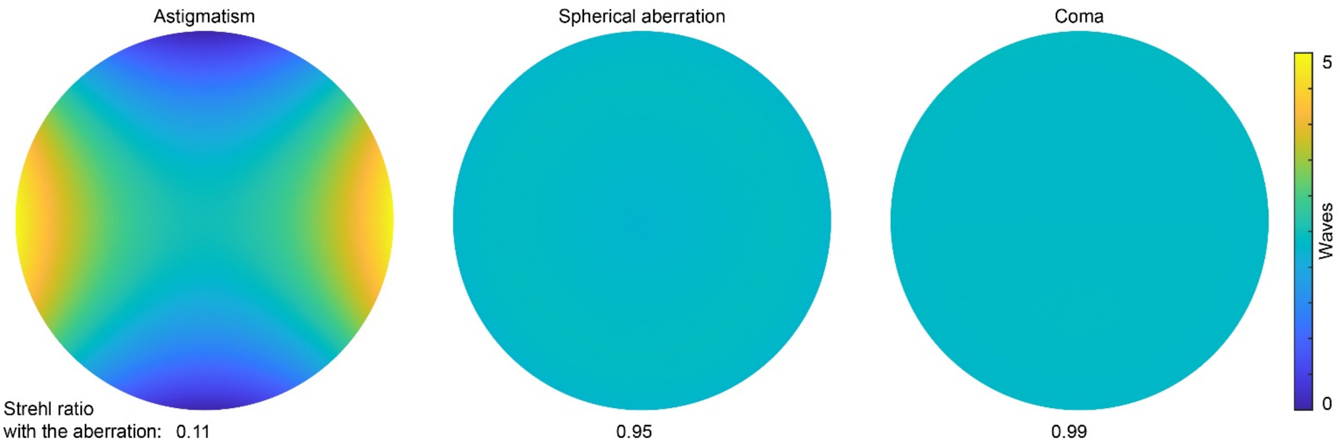

Supplementary Figure 2 | Aberration through 0.5 mm diameter 1.5 pitch GRIN lens. (a) The Zernike coefficients for the aberration shown in Fig. 1a. (b) The wavefront and the corresponding Strehl ratio of astigmatism, spherical aberration, and coma.

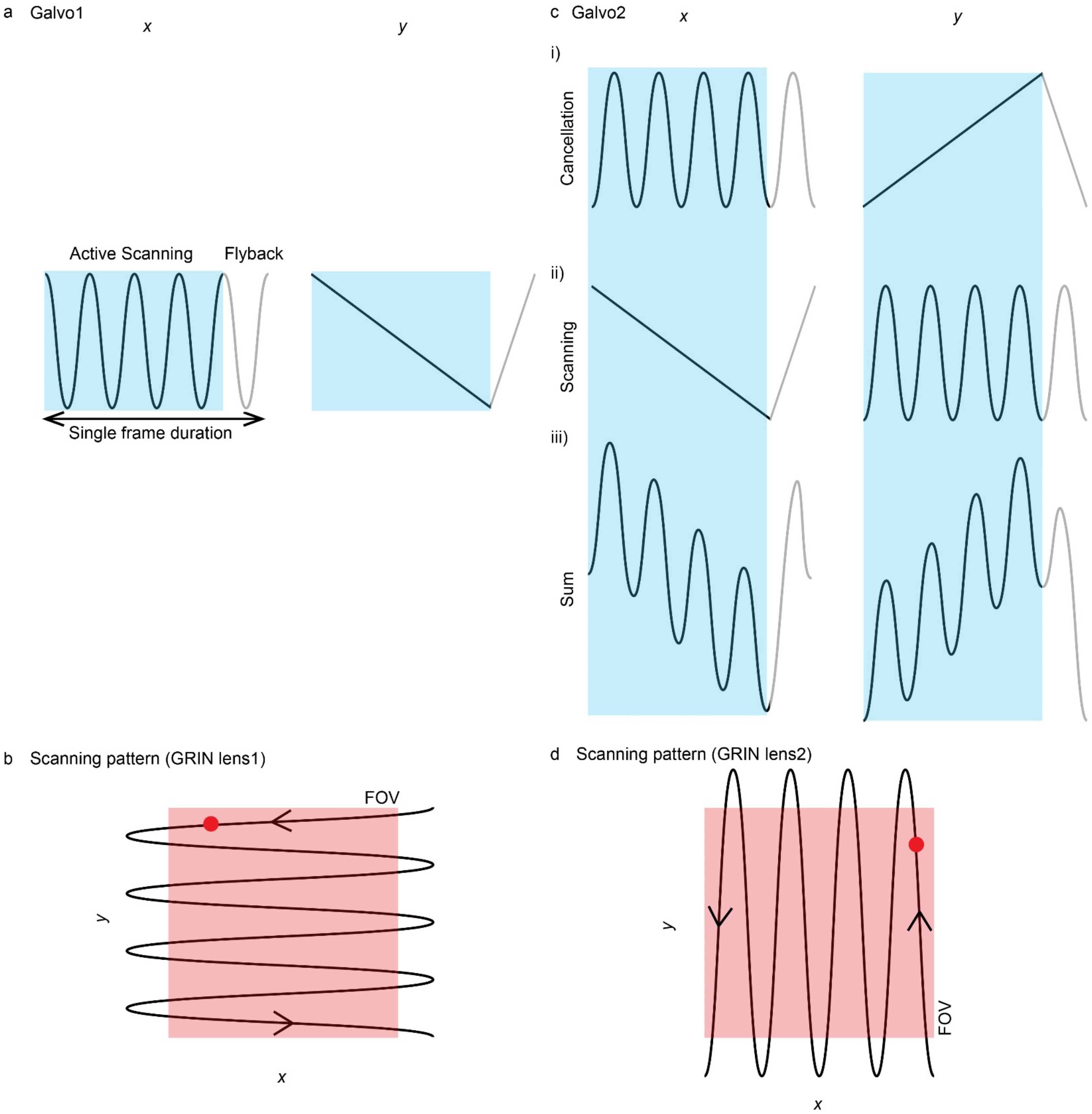

Supplementary Figure 3 | Galvo control signal for achieving the 90-degree rotation. (a) Galvo 1 control signals for *x* and *y* axis. (b) The laser scanning path on the facet of GRIN lens 1. (c) Galvo 2 control signals for *x* and *y* axis. (d) The laser scanning path on the facet of GRIN lens 2.

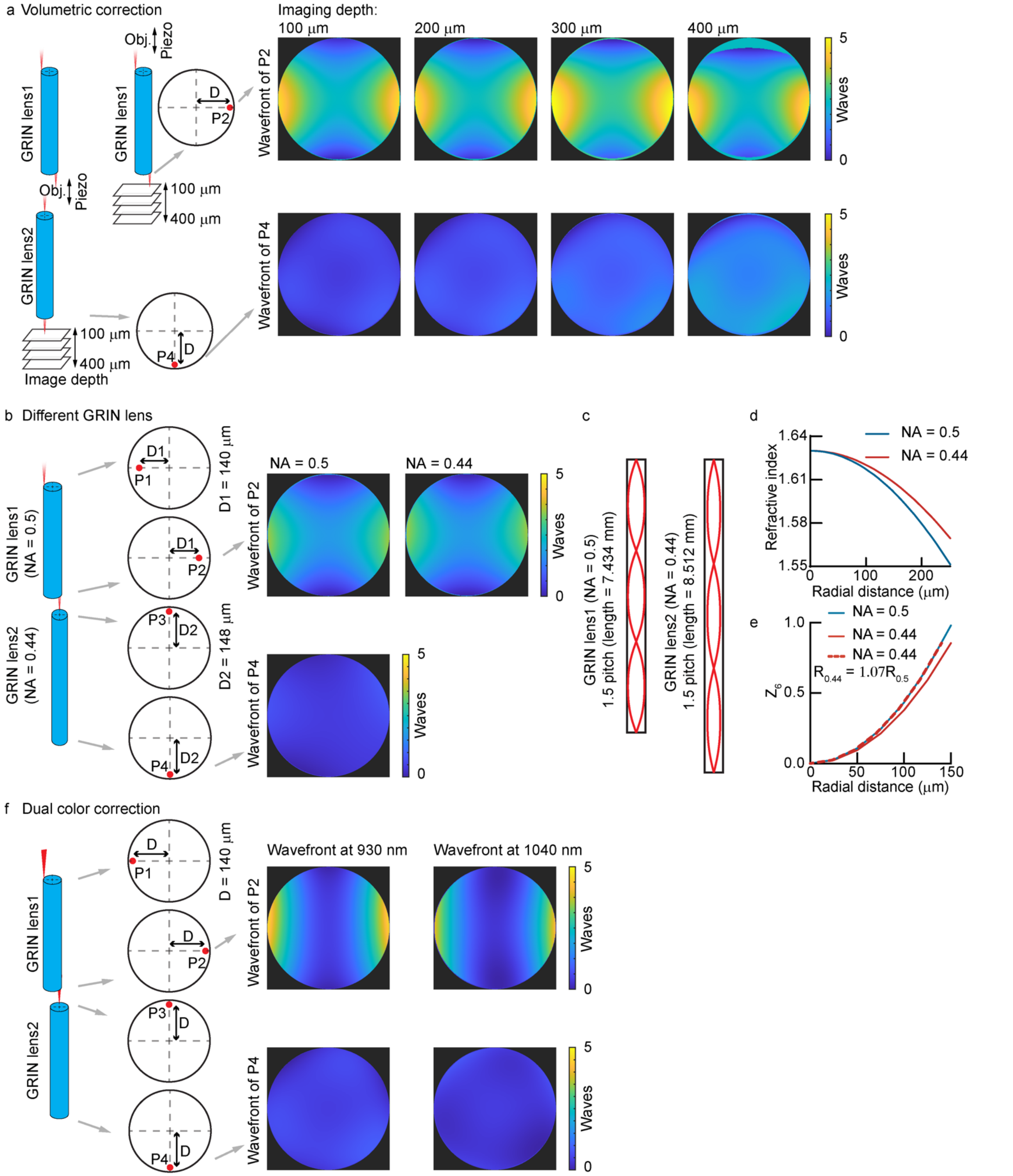

Supplementary Figure 4 | Zemax simulation. (a) Configuration for volumetric correction and the Zemax calculated wavefront profiles. (b) Configuration for using GRIN lens of maximum NA 0.5 to correct GRIN lens of maximum NA 0.44 and the Zemax calculated wavefront profiles. (c, d) The corresponding optical paths and refractive index profiles inside GRIN lenses. (e) Aberration as a function of field position. With a position ratio of 1.07:1, the astigmatism of the NA 0.44 lens can precisely cancel that of the NA 0.5 lens over the entire FOV. (f) Configuration for dual-color correction (GTAO calibrated for 930 nm) and the Zemax calculated wavefront profiles.

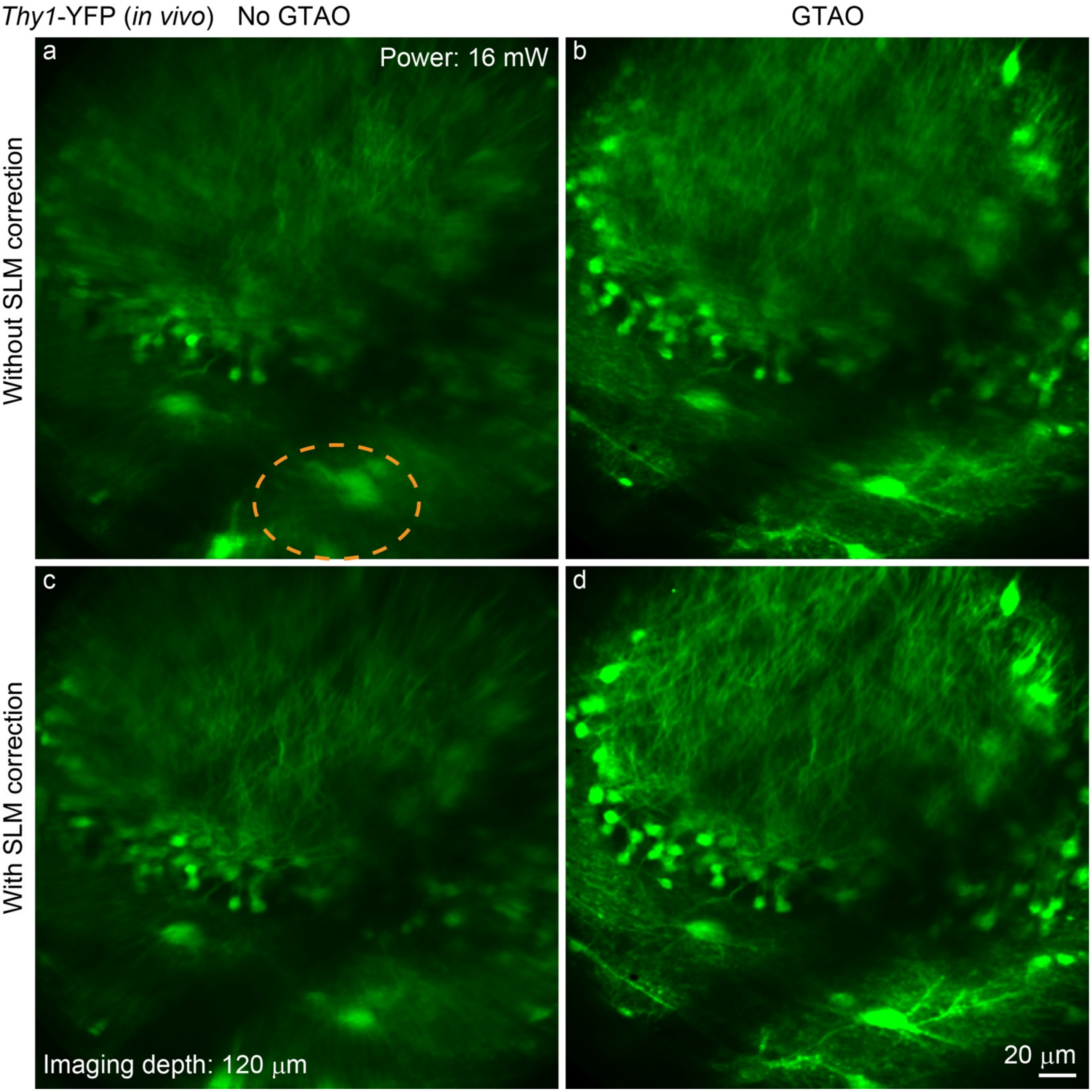

Supplementary Figure 5 | Evaluate on-axis correction for *in vivo* structural imaging. (a) *In vivo* structural imaging without GTAO or SLM correction (flat wavefront after the SLM). (b) Imaging with GTAO but without SLM correction. (c) Imaging without GTAO but with SLM correction. (d) Imaging with both GTAO and SLM correction. Overall, the neurons encircled by the dashed line were only visible with GTAO regardless of the usage of SLM. The imaging experiment was repeated independently 2 times with similar results.

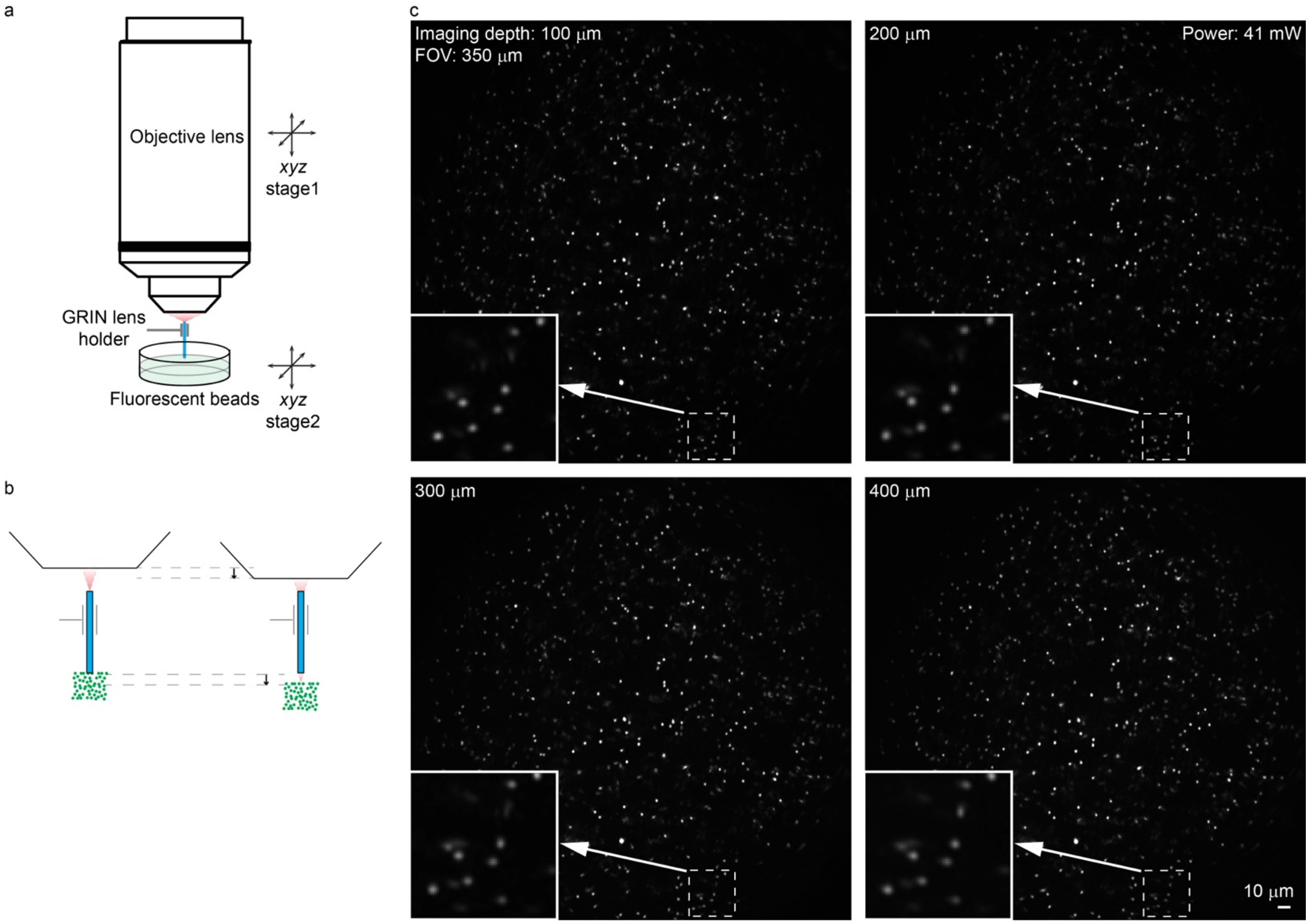

Supplementary Figure 6 | Volumetric correction and imaging through 0.5 mm diameter 1.5 pitch GRIN lens. (a) Experiment configuration. (b) The distances between the GRIN lens, the objective lens, and the sample were adjusted so that the same sample region was in focus at different working distances (100-400 microns). (c) The images recorded with the same galvo scanning control signals. The imaging experiment was repeated 1 time.

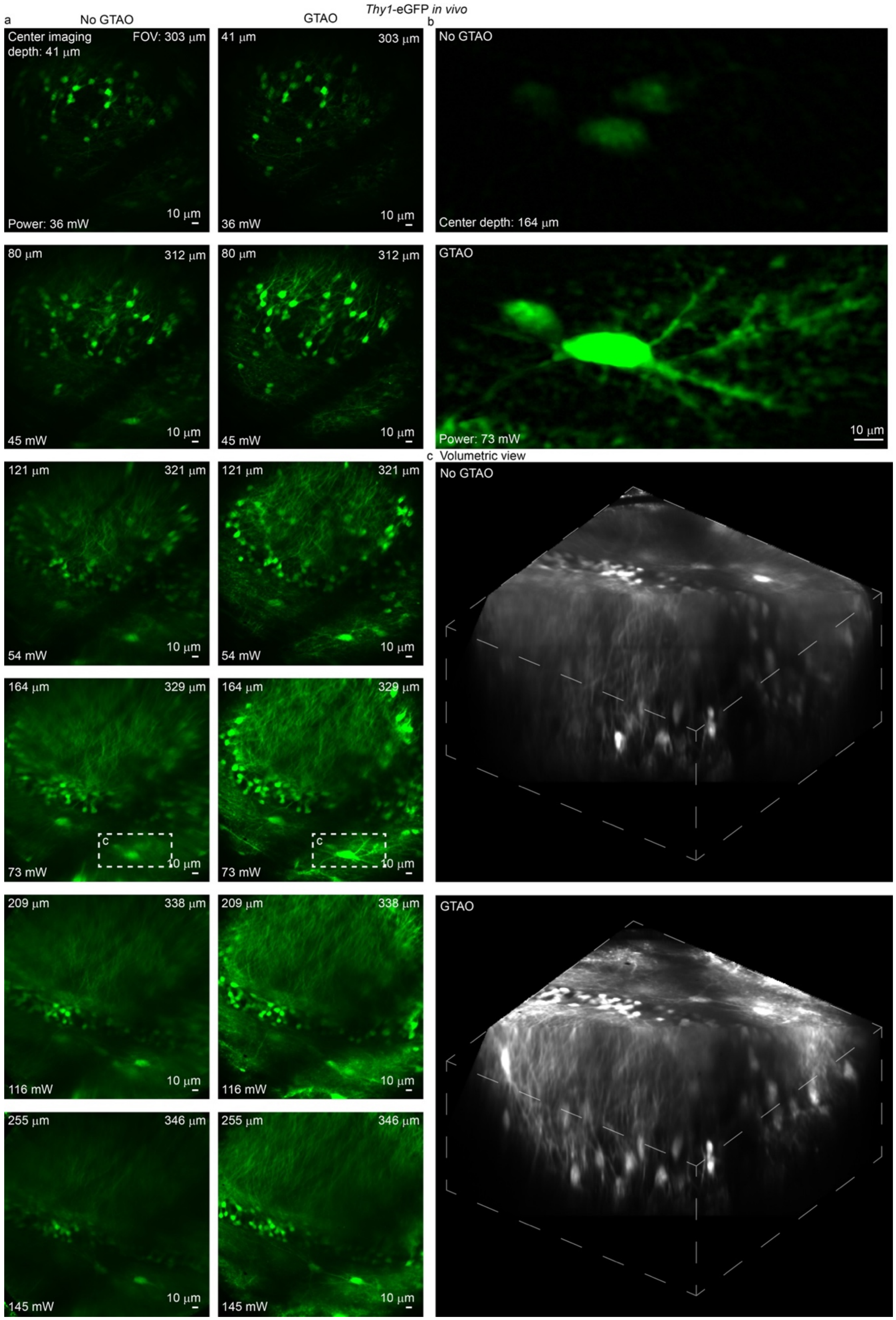

Supplementary Figure 7 | *In vivo* volumetric imaging through 0.5 mm diameter 1.4 pitch GRIN lens. (a) Image comparison at different WD. (b) Zoomed-in view of the dashed box. (c) Comparison of rendered image volumes. The imaging experiment was repeated independently 2 times with similar results.

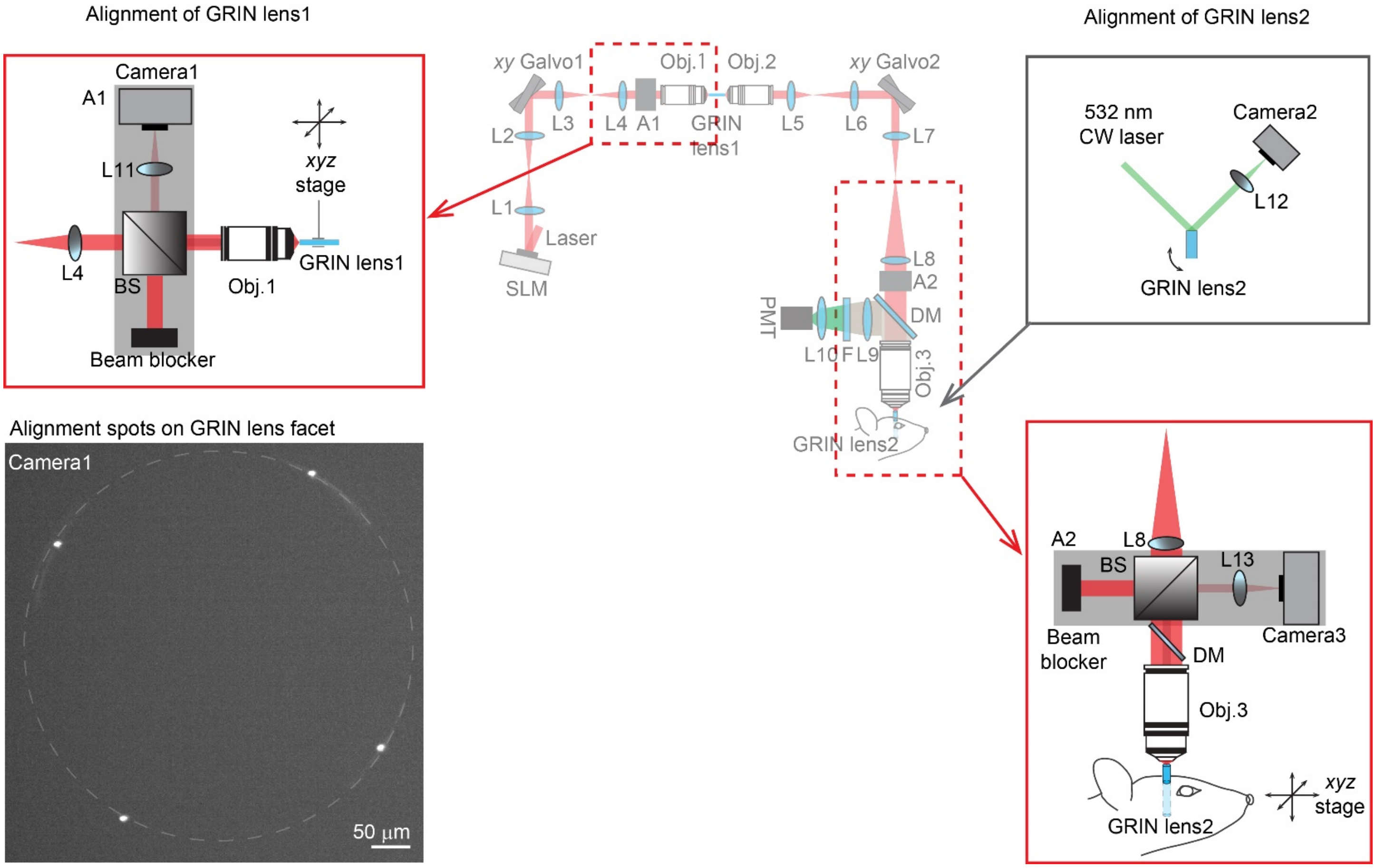

Supplementary Figure 8 | Optical setup for alignment and calibration of GTAO. A1 and A2 show the locations of magnetic mounts that allow the insertion and removal of optical beam splitters. Through the beam splitters, Cameras 1 and 3 can visualize the facets of GRIN lenses for precise position alignment. To center the GRIN lens, we employed the scanner to position the focus on four locations and adjusted the GRIN lens position such that the four spots were right on the edge of the GRIN lens facet. Camera 2 with lens L12 showed the orientation of the GRIN lens implanted inside the mouse brain.
