# Supplementary material for: Geometric transformation adaptive optics (GTAO) for volumetric deep brain imaging through gradient-index lenses
Source: Nat Commun. 2024 Feb 3;15:1031. doi: 10.1038/s41467-024-45434-5 (PMC10838304; doi:10.1038/s41467-024-45434-5)
Supplement: Supplementary file 3 — Description of Additional Supplementary Files [file 41467_2024_45434_MOESM3_ESM.pdf]

Description of Additional Supplementary Files:

Supplementary Movie 1: Volumetric imaging of neurons in brain slice through a 0.5 mm diameter 1.5 pitch GRIN lens.

Supplementary Movie 2: In vivo volumetric recording of neurons in mouse brain through a 0.5 mm diameter 1.5 pitch GRIN lens.

Supplementary Movie 3: In vivo 2D calcium recording through a 0.5 mm diameter 1.5 pitch GRIN lens.

Supplementary Movie 4: In vivo 3D volumetric calcium recording using GTAO through a 0.5 mm diameter 1.5 pitch GRIN lens.
