# Supplementary material for: Geometric transformation adaptive optics (GTAO) for volumetric deep brain imaging through gradient-index lenses
Source: Nat Commun. 2024 Feb 3;15:1031. doi: 10.1038/s41467-024-45434-5 (PMC10838304; doi:10.1038/s41467-024-45434-5)
Supplement: Supplementary file 8 — Reporting Summary [file 41467_2024_45434_MOESM8_ESM.pdf]

nature portfolio

Corresponding author(s): Meng Cui
Last updated by author(s): 01/03/2024

Reporting Summary

Nature Portfolio wishes to improve the reproducibility of the work that we publish. This form provides structure for consistency and transparency in reporting. For further information on Nature Portfolio policies, see our Editorial Policies and the Editorial Policy Checklist.

Statistics

For all statistical analyses, confirm that the following items are present in the figure legend, table legend, main text, or Methods section.

n/a	Confirmed	
☐	☒	The exact sample size (*n*) for each experimental group/condition, given as a discrete number and unit of measurement
☐	☒	A statement on whether measurements were taken from distinct samples or whether the same sample was measured repeatedly
☐	☒	The statistical test(s) used AND whether they are one- or two-sided<br>*Only common tests should be described solely by name; describe more complex techniques in the Methods section.*
☒	☐	A description of all covariates tested
☒	☐	A description of any assumptions or corrections, such as tests of normality and adjustment for multiple comparisons
☒	☐	A full description of the statistical parameters including central tendency (e.g. means) or other basic estimates (e.g. regression coefficient) AND variation (e.g. standard deviation) or associated estimates of uncertainty (e.g. confidence intervals)
☐	☒	For null hypothesis testing, the test statistic (e.g. *F*, *t*, *r*) with confidence intervals, effect sizes, degrees of freedom and *P* value noted<br>*Give P values as exact values whenever suitable.*
☒	☐	For Bayesian analysis, information on the choice of priors and Markov chain Monte Carlo settings
☒	☐	For hierarchical and complex designs, identification of the appropriate level for tests and full reporting of outcomes
☒	☐	Estimates of effect sizes (e.g. Cohen's *d*, Pearson's *r*), indicating how they were calculated

Our web collection on statistics for biologists contains articles on many of the points above.

Software and code

Policy information about availability of computer code

Data collection	Scanimage 5.6 was used for data collection.
Data analysis	GraphPad Prism (10.1.0) and MATLAB 2021a were used for statistical analysis.

For manuscripts utilizing custom algorithms or software that are central to the research but not yet described in published literature, software must be made available to editors and reviewers. We strongly encourage code deposition in a community repository (e.g. GitHub). See the Nature Portfolio guidelines for submitting code & software for further information.

Data

Policy information about availability of data

All manuscripts must include a data availability statement. This statement should provide the following information, where applicable:
- Accession codes, unique identifiers, or web links for publicly available datasets
- A description of any restrictions on data availability
- For clinical datasets or third party data, please ensure that the statement adheres to our policy

The data collected and analyzed in this study have been uploaded to the Science Data Bank database.

Research involving human participants, their data, or biological material

Policy information about studies with human participants or human data. See also policy information about sex, gender (identity/presentation), and sexual orientation and race, ethnicity and racism.

Reporting on sex and gender	not applicable
Reporting on race, ethnicity, or other socially relevant groupings	not applicable
Population characteristics	not applicable
Recruitment	not applicable
Ethics oversight	not applicable

Note that full information on the approval of the study protocol must also be provided in the manuscript.

Field-specific reporting

Please select the one below that is the best fit for your research. If you are not sure, read the appropriate sections before making your selection.

- [x] Life sciences
- [ ] Behavioural & social sciences
- [ ] Ecological, evolutionary & environmental sciences

For a reference copy of the document with all sections, see nature.com/documents/nr-reporting-summary-flat.pdf

Life sciences study design

All studies must disclose on these points even when the disclosure is negative.

Sample size	The imaging experiments were repeated on different mice to adequately demonstrate the imaging capabilities of GTAO. All imaging experiments were repeated on at least 4 mice. These measurement on different animals yielded consistent results, and the sample size was sufficient to support the conclusions of this work.
Data exclusions	No data exclusion.
Replication	Each type of animal experiment was repeated on at least 3 mice, which showed consistent results.
Randomization	In this study, there was no distinction between a control group and an experimental group; instead, the focus was to compare the differences in the imaging system with and without GTAO. The imaging experiments involved both male and female mice, and there were no significant differences in imaging outcomes between the genders. Randomization was not pertinent to this study, as the optical imaging performance was independent of the allocation of animals.
Blinding	No blinding was used as the imaging performance was independent of animals. There were no control group and experiment group distinctions in this work.

Reporting for specific materials, systems and methods

We require information from authors about some types of materials, experimental systems and methods used in many studies. Here, indicate whether each material, system or method listed is relevant to your study. If you are not sure if a list item applies to your research, read the appropriate section before selecting a response.

Materials & experimental systems

n/a	Involved in the study	
x		Antibodies
x		Eukaryotic cell lines
x		Palaeontology and archaeology
	x	Animals and other organisms
x		Clinical data
x		Dual use research of concern
x		Plants

Methods

n/a	Involved in the study	
x		ChIP-seq
x		Flow cytometry
x		MRI-based neuroimaging

Animals and other research organisms

Policy information about studies involving animals; ARRIVE guidelines recommended for reporting animal research, and Sex and Gender in Research

Laboratory animals	Wild-type C57BL/6 mice and Thy1-eGFP M line mice were purchased from the Jackson Laboratory. These mice were accommodated at Bindley Bioscience Center's animal facility. Surgical work was carried out on both adult male and female mice, aged between two and four months. The facility maintained a light cycle of 12 hours on and off, with lights on from 6 am to 6 pm. The environmental conditions were carefully controlled, with temperatures sustained at 20-25 degrees Celsius.
Wild animals	No wild animals were used.
Reporting on sex	The imaging experiment used both male and female mice, and the experimental results were independent of gender.
Field-collected samples	No field-collected samples were used.
Ethics oversight	All procedures involving mice were approved by the Purdue University Animal Care and Use Committee (protocol number: 1506001267).

Note that full information on the approval of the study protocol must also be provided in the manuscript.

Plants

Seed stocks	not applicable
Novel plant genotypes	not applicable
Authentication	not applicable
